# Determination of adjusted reference intervals of urinary biomarkers of oxidative stress in healthy adults using GAMLSS models

**DOI:** 10.1371/journal.pone.0206176

**Published:** 2018-10-23

**Authors:** Liliya Chamitava, Vanessa Garcia-Larsen, Lucia Cazzoletti, Paolo Degan, Andrea Pasini, Valeria Bellisario, Angelo G. Corsico, Morena Nicolis, Mario Olivieri, Pietro Pirina, Marcello Ferrari, Mikis D. Stasinopoulos, Maria E. Zanolin

**Affiliations:** 1 Unit of Epidemiology and Medical Statistics (SESM), Department of Diagnostics and Public Health, University of Verona, Verona, Italy; 2 Program in Human Nutrition, Department of International Health, The Johns Hopkins Bloomberg School of Public Health, Baltimore, United States; 3 Epidemiology, Prevention and Special Functions, National Institute of Cancer Research AOU S. Martino IST, Genova, Italy; 4 Department of Internal Medicine, University of Verona, Verona, Italy; 5 Department of Public Health and Pediatrics, University of Turin, Torino, Italy; 6 Division of Respiratory Diseases, ERCS, S. Matteo, Hospital University of Pavia, Pavia, Italy; 7 Unit of Hygiene and Preventive, Environmental and Occupational Medicine, Department of Diagnostics and Public Health, University of Verona, Verona, Italy; 8 Unit of Occupational Medicine, University of Verona, Verona, Italy; 9 Institute of Respiratory Diseases, University of Sassari, Piazza Università, Sassari, Italy; 10 Department of Medicine, Unit of Respiratory Medicine, University of Verona, Verona, Italy; 11 Statistics, Operational Research and Mathematics Research Centre, London Metropolitan University, London, United Kingdom; International University of Health and Welfare, School of Medicine, JAPAN

## Abstract

In this study we aimed at identifying main demographic, laboratory and environmental factors influencing the level of urinary biomarkers (DNA-derived 8-oxodG and lipid membrane-derived 8-isoprostane), and deriving their adjusted 95% reference intervals (RI) in a sample of healthy people from the general population. Data from 281 healthy subjects from the Gene Environment Interactions in Respiratory Diseases survey were used in this study. Generalized additive models for location, scale and shape (GAMLSS) were used to find determinants of the biomarkers among gender, age, season and distance from collection (DFC), and to predict their RI. The RI of the biomarkers stratified by season and adjusted for DFC showed a slight statistically significant decrease in the biomarkers at the increasing DFC in two seasons, except the 8-oxodG during the warm season: median levels at the min and max values of DFC were (ng/mgcreat) 7.0–1.1 in the cold and 3.9–3.9 in the warm seasons for 8-oxodG, 0.7–0.2 in the cold and 1.3–0.6 in the warm seasons for 8-isoprostane. Both the biomarkers should be evaluated in association with the DFC and season in large epidemiological studies. The (semi)parametric GAMLSS method is a useful and flexible technique, which makes it possible to estimate adjusted RI.

## Introduction

Oxidative stress (OS) is a central feature in the molecular pathways leading to the expression of many chronic and acute diseases [1]. OS is defined as an imbalance between the production of reactive oxygen species (ROS, which are natural by-products of oxygen metabolism) and the antioxidant defensive capacity of an organism [2, 3]. Excessive production of ROS has been demonstrated to suppress antioxidant capacity *in vivo*, damaging DNA, lipids, and proteins [4]. The highly reactive nature of ROS and, in turn, their short half-life make their measurement difficult. Instead, measures of OS usually rely on the assessment of the products of oxidising reactions, with 8-oxodG (8-oxo-7,8-dihydro-2’-deoxyguanosine) and 8-isoprostane (8-iso-prostaglandin F_2α_) being two of the most commonly used DNA- and lipid-derived biomarkers of OS, respectively [5, 6].

The stability of 8-oxodG concentrations in urine makes this molecule a natural choice of OS marker [7]. The 8-isoprostane is considered the main biomarker of oxidative catalysis of arachidonic acid *in vivo* [8]. Although urine is a reliable fluid to measure concentration of these two biomarkers [7, 9], little is known about the external factors that can influence variations in their urinary measurements. Using data on healthy adults collected within a population-based study, we aimed at identifying the demographic, environmental and laboratory-based determinants of variations in concentration of 8-oxodG and 8-isoprostane in urine, and to build adjusted reference intervals (RIs) using a novel, flexible statistical approach: the Generalized Additive Models for Location, Scale and Shape (GAMLSS). This regression technique makes assumptions on a distribution form and provides a variety of different distribution families for the response variable; and has a platform to fit, compare and check many different models [10]. GAMLSS model the distribution parameters: μ (a location parameter, i.e. mean, median), σ (a scale, i.e. standard deviation, dispersion), ν (a shape, i.e. modelling skewness) and τ (a shape, i.e. modelling kurtosis) [11]. It is possible to fit with GAMLSS additive or the multiplicative models for μ using identity or log links respectively [12]. The WHO has adopted the GAMLSS methodology for creating reference growth curves [13].

## Materials and methods

### Study sample

The GEIRD survey was set up to investigate the association of several risk factors for chronic respiratory disease. Full details are available elsewhere [14]. In brief, between 2007 and 2013, adults aged 20–64 years old were randomly selected from sampling frames in four Italian centres in the general population of Verona, Pavia, Turin, and Sassari [14, 15]. Data on respiratory symptoms, lung function spirometry, and related risk factors were collected. Participants were subsequently invited to undergo detailed clinical interview and tests for accurate phenotyping, and to provide blood and urine samples [14, 16].

Out of 16569 subjects selected to participate in GEIRD stage 1, 9741 (59%) answered the screening questionnaire, 4981 (51%) of them were selected to attend the GEIRD stage 2 and 2259 (45%) participated in the clinical survey. The current analyses were restricted to 281 subjects who reported no respiratory conditions, had normal lung function test [14, 17] and who declared to be ex- or never smokers.

### Clinical and laboratory measurements

#### Urine collection

Participants were asked to collect a spot quantity of the first morning urine in a clean container, as well as to indicate the time when it was gathered, and to declare the number of cigarettes smoked and medicines taken before, if any [14].

The container with the urine sample was stored at 4°C over 24 hours. Then equal rates of 1 ml were derived and frozen at -80°C pending further laboratory examination.

#### Biomarkers evaluation

The 8-oxodG (8-oxo-7,8-dihydro-2’-deoxyguanosine) and the 8-isoprostane (8-iso-prostaglandin F_2α_), both standardized by creatinine (ng/mg), were evaluated with the immunosorbent assay kit ELISA (Cosmo Bio LTD, Tokyo Japan, and Cayman Chemical, Ann Arbor, MI, USA, respectively). Another ELISA kit (Cayman Chemical, Ann Arbor, MI, USA) was used for the assessment of creatinine concentration (mg/ml).

The laboratory processing of the 8-oxodG concentrations was started after 36 days in the cold season and 148 days in the warm season of urine storage. The laboratory processing of the 8-isoprostane concentrations was started after 265 days in the cold season and 230 days in the warm season of urine storage.

All urine samples were analyzed in the laboratory of Genova (Epidemiology, Prevention and Special Functions, National Institute of Cancer Research AOU S. Martino IST, Genova, Italy).

### Statistical analyses

The semi-parametric GAMLSS (generalized additive models for location, scale and shape) in R (Version 0.99.902–2009–2016 RStudio, Inc.) regression models were used to analyze the association of several factors and the concentration of urinary 8-oxodG and 8-isoprostane [18, 19].

GAMLSS were used to answer the following questions:

1) Are all explanatory variables (e.g. age, gender, etc.) required in the model?
2) Is the relationship between the chosen covariates additive or multiplicative? For example identify whether log or identity link functions should be used for modelling the location parameter.
3) What type of relationship does exist between the parameters and the explanatory variables: linear, quadratic or more complex?
4) Is the final model adequate for the data?

Four exposures related to the participant, laboratory processing, and collection of sample (age, gender, distance from collection (DFC, i.e. the period from the moment of urine collection and its laboratory processing), and season (the period of a year split into warm (*April—September*) and cold (*October—March*)) were examined for their association with the two biomarkers of interest using multivariable GAMLSS regression models. The fitting of GAMLSS models in entire 8-oxodG and 8-isoprostane samples was performed to answer the question n.1, i.e. to identify which of the 4 covariates should be included in the model. According to the obtained results, all models were stratified by season and adjusted for DFC.

The development of these models was first performed in the framework of each distribution family, i.e. the Box-Cox Cole and Green (BCCG), gamma (GA) and log-normal (LOGNO), applying polynomials, cubic and penalized B-splines (P-splines) [20], to the mean (median) μ, variability σ skewness ν, and kurtosis τ [11, 19, 21]. The best model for each biomarker was chosen by minimizing the Bayesian information criterion (BIC) [22] and by checking the normalized quantile residuals. The simplicity of the model and biologically plausible centile curves were also taken into account.

Outliers for each biomarker were identified using Influence (bubble) plots [23]. In the final step, GAMLSS models for each biomarker per each season were fitted by: i) excluding outliers, ii) using cubic splines, iii) simultaneously by excluding outliers and using cubic splines. The `best’ models excluded outliers and used only linear terms; these models were later used for building reference intervals of the 8-oxodG and 8-isoprostane as functions of the DFC in two seasons.

## Results

Table 1 illustrates the main characteristics of the participants (n = 281) with available information on 8-oxodG (n = 275) and on 8-isoprostane (n = 227). The mean age of the sample was 44.5 years and 57% were women. The median distance from sample collection (DFC) was 387 days, with half of the samples drawn during the warm season.

**Table 1:** Characteristics of the sample.

RESPONSE VARIABLES	n	MEDIAN (IQR)	MEAN(SD)	RANGE
**8-oxodG,** ng/mg_*creat*_	275*	3.89 (1.91–7.95)	6.75 (10.60)	(0.06–108.00)
**8-isoprostane,** ng/mg_*creat*_	227*	0.60 (0.24–1.57)	1.09 (1.30)	(0.00–9.77)
**EXPLANATORY VARIABLES**				
**Age,** years	281	44.80 (38.46–51.11)	44.51 (9.23)	(22.51–65.83)
**DFC,** days	255*	387 (316–611)	433.59 (190.87)	(36–765)
**Gender,** females (%)	161(57%)	-	-	-
**Season,** warm (%)	127(50%)*	-	-	-

*Out of 281 selected subjects 6 did not have data on the 8-oxodG, 54 on the 8-isoprostane, 26 on the DFC and Season. DFC–Distance From Collection—the period from the moment of urine collection and its laboratory processing; Season–warm (April—September), cold (October—March).

A preliminary analysis was performed to identify the important explanatory variables. A selection using BIC within GAMLSS indicate that the DFC is important for both the 8-oxodG and 8-isoprostane responses during the cold and warm seasons for all the parameters of the fitted distributions. These models were identified as ‘Standard’ (S1 Table). Model diagnostics were used to identify possible influential observations.

As a final step, models, eliminating influential observations, fitting cubic splines to identify nonlinear relations or both of those alternatives, were tried and compared to the previously best fitted “Standard” models. Note that the models with cubic splines were fitted to avoid influential extreme values in the outcome variables, however did not improve the fit for both biomarkers in both seasons. The `best’ models were chosen according to the smallest BIC, adequacy of the residuals and parsimony (S1 Table).

Table 2 (models 1 and 2) shows that the `best’ final models of the 8-oxodG during both the cold and warm seasons were the log-normal (LOGNO) distribution models with a log link for both the mu and sigma. The BCCGo distribution model provided the best fitting for the 8-isoprostane in the cold season. This model had log link for mu and sigma and identity link for the skewness parameter nu. The Gamma distribution model was selected for the 8-isoprostane in the warm season. In all the models above the influential observations were excluded while the resulting residual proved to be adequate (Table 2 and S1, S2, S3 and S4 Figs).

**Table 2:** The best GAMLSS models for the 8-oxodG and 8-isoprostane during the cold and warm seasons with linear predictors for median μ, variability σ and skewness ν.

Distribution	Linear predictor for μ			Linear predictor for log σ		Linear predictor for ν			df	BIC
	Link Function	Edf_μ*DFC*_	p_*DFC*_	edf_σ*DFC*_	p_*DFC*_	Link Function	edf_ν*DFC*_	p_*DFC*_		
**8-oxodG. Cold season.**										
**1. LOGNO**	log	0	linear	0	linear	-	-	-	4	627.02
**8-oxodG. Warm season.**										
**2. LOGNO**	log	0	linear	0	-	-	-	-	3	649.96
**8-isoprostane. Cold season.**										
**3. BCCGo**	log	0	linear	0	linear	identity	0	linear	4	166.32
**8-isoprostane. Warm season.**										
**4. GA**	log	0	linear	0	-	-	-	-	3	217.49

pDFC—polynomials or splines fitted in a GAMLSS (generalized additive models for location, scale and shape) formula. edf–effective degrees of freedom; characterize the complexity of spline curves; edf = 0 corresponds to the linear term, the higher it is, the more complex is the curve [12]. Distribution families: LOGNO—log-normal; BCCGo–Box-Cox Cole and Green; GA–Gamma.

**Fig 1:**
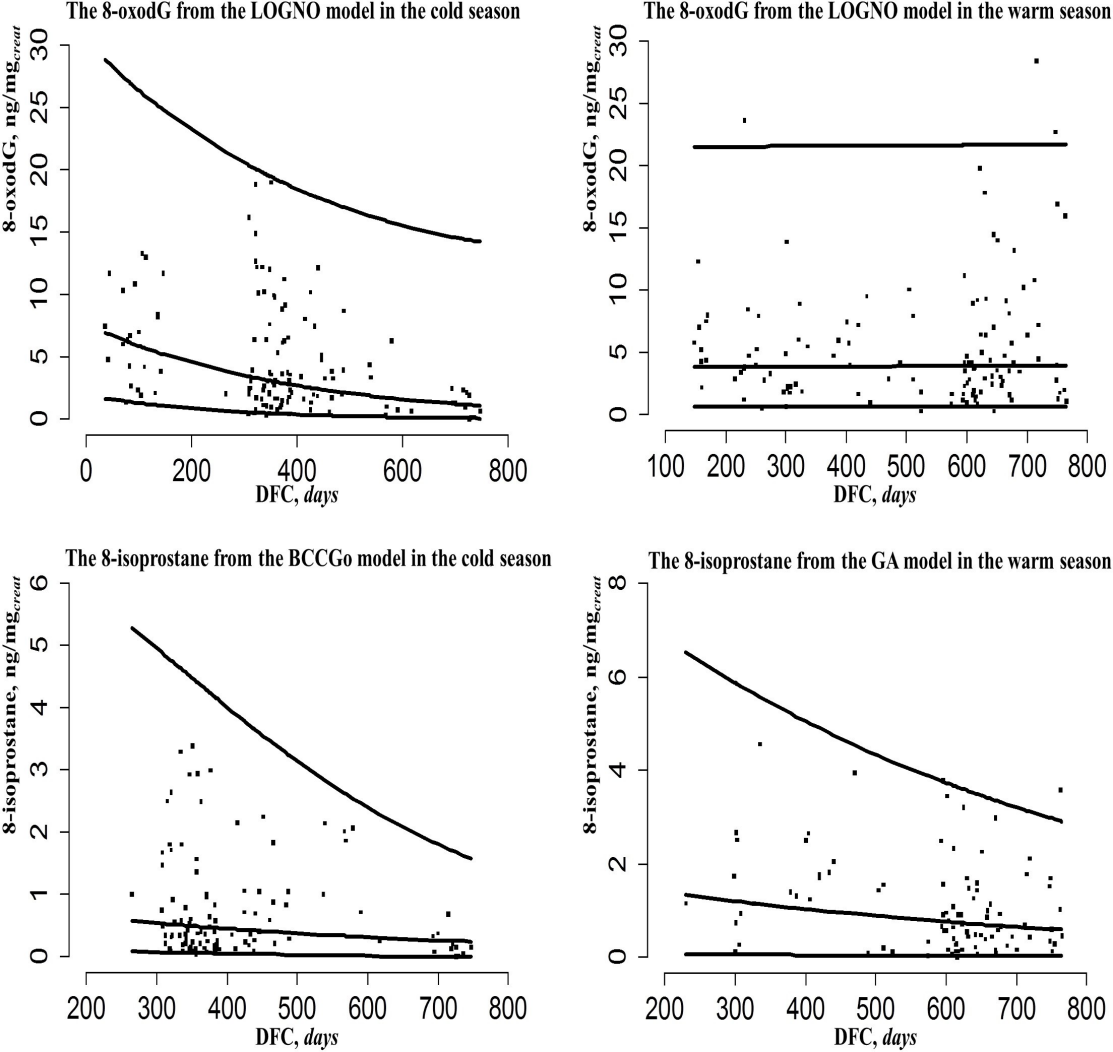
The observed 8-oxodG and 8-isoprostane values with three fitted model centile curves (2.5%, 50%, 97.5%) against the DFC in two seasons. Three values of the 8-oxodG ng/mg_*creat*_ and 8-isoprsotane ng/mg_*creat*_ at the lowest, median and highest values of the distance from collection (DFC) are shown on the median (50%) centile curves of each biomarker in each season. Distribution families: LOGNO—log-normal; BCCGo–Box-Cox Cole and Green; GA–Gamma.

Fig 1 shows the 95% reference (2.5%, 97.5%) and median (50%) curves calculated using the best developed model for each biomarker (8-oxodG and 8-isoprostane) in two seasons against the distance from collection (DFC). The RI of the biomarkers stratified by season and adjusted for DFC showed a slight but statistically significant median decrease in the biomarkers at the increasing DFC in two seasons, except the 8-oxodG during the warm season: median levels at the min and max values of DFC were 7.0–1.1 ng/mg_*creat*_ in the cold and 3.9–3.9 ng/mg_*creat*_ in the warm seasons for 8-oxodG, 0.7–0.2 ng/mg_*creat*_ in the cold and 1.3–0.6 ng/mg_*creat*_ in the warm seasons for 8-isoprostane.

During the cold season (Fig 1 and S2 Table), the 8-oxodG values decreased from 1.68 to 0.09 ng/mg_*creat*_ for the lower 2.5% limit of 95% RI and from 28.85 to 14.33 ng/mg_*creat*_ for the upper 97.5% limit of 95% RI. During the warm season (Fig 1 and S3 Table), the 8-oxodG values were quite constant: 95% RI = 0.71–21.55 ng/mg_*creat*_ at the beginning of the urinary 8-oxodG measuring and 95% RI = 0.71–21.75 ng/mg_*creat*_ at the end of its measuring. There was also a decrease of the 8-isoprostane values during the cold season (Fig 1 and S4 Table): 95% RI = 0.05–4.77 ng/mg_*creat*_ at the beginning of urinary 8-isoprostane measuring and 95% RI = 0.02–1.60 ng/mg_*creat*_ at the end of its measuring. During the warm season (Fig 1 and S5 Table) the 8-isoprostane values decreased as well: 95% RI = 0.06–6.54 ng/mg_*creat*_ at the beginning and 95% RI = 0.03–2.93 ng/mg_*creat*_ at the end.

## Discussion

Generally, it is considered that oxidative stress is associated with aging [24]. Sakano N. et al. [6] found a statistically significant difference in the 8-oxodG level in two healthy Japanese age groups: the concentration of the urinary 8-oxodG in persons who were over 40 years old was significantly higher than that in persons who were under 40. The study carried out by Ogino K et al. showed that the urinary 8-oxodG had significantly higher level in persons over 45 years old compared with the level in persons under 45 years old. [25]. Topic et al. found that the 8-oxodG values were lower in younger subjects than in older [26]. We did not found a significant difference either in the 8-oxodG, or in the 8-isoprostane values associated with age in our study. A group of scientists from Parma [27] investigated healthy Italian subjects and did not find a significant association of the 8-oxodG values with age as well. Kimura et al. showed that mean urinary 8-oxodG was not significantly different in terms of age in healthy Japanese people [28]. An absence of a significant association of the 8-isoprostane with age was found also by Sakano et al. [6] and Ogino et al. [25].

There are also inconsistent data on OS biomarker values in gender-related subpopulations. We did not find differences in the 8-oxodG and 8-isoprostane values associated with gender in our study. Andreoli et al. [27], Topic et al. [26] and Sakano et al [6] did not find gender-related differences in the 8-oxodG values either. While Lily Wu et al. [1] showed that normal values of the 8-oxodG in females were higher than in males. On the other hand, K. Oginoet al. found that men had a significantly higher level of the 8-oxodG in respect to women [25], whereas they showed that the 8-isoprostane values were independent of gender. Meanwhile, Sakano et al. found higher mean values of the 8-isoprostane in men in respect to women [6].

To our knowledge, there are no studies analyzing the association between the 8-oxodG and 8-isoprostane and the season of urine collection apart from the studies conducted by Rossner et al. in which they investigated the seasonal variability of the 8-oxodg and 8-isoprostane in bus drivers in comparison to controls affected by environmental pollutants [29, 30]. Some studies on the clinical examination of the concentration change of the OS biomarkers in urine with time [7, 31, 32] were not performed in an epidemiological setting, as in the current study, which included the variable such as the DFC. In our study, the season and DFC proved to be influential on values of both the biomarkers, and hence they should be considered when constructing the reference intervals of both the OS biomarkers. These variables can be very important in large longitudinal epidemiological studies, which could require a longer time for urine conservation before its laboratory analysis. Environmental factors, such as the season when the urine was collected can influence the biomarker values due to the seasonal differences in human metabolism, air temperatures, higher exposure to UV radiation, environmental pollutants [29, 30] etc.

Some studies report a high stability of the 8-oxodG in urine [7, 31, 32], but there is also inconsistent data on whether its level decreases or increases with time. Thus, Y. Matsumoto et al. stated that the concentration of the urinary 8-oxodG can increase during a long period of conservation because of progressive DNA oxidation [31]. M. Nakajima et al. found that oxygen molecules in the environment can provoke accidental ROS generation and can consequently lead to an additional 8-oxodG formation in urine samples [33]. In the meantime, Y. Matsumoto et al. found that 8-oxodG remains stable in urine for over two years if stored at -80°C [31]. Shigenaga et al. [34] showed that there is no additional formation of the 8-oxodG when urine was stored at 4°C for 19 days. In our study, we found a slight but significant decrease of the 8-oxodG with time in the urine that was collected during the cold season. The concentration of the 8-oxodG remained stable over the entire period of storage when urine was collected during the warm season. This could be explained by an additional formation of the 8-oxodG in urine during the warm season (April—September) if what Y. Matsumoto et al. and M. Nakajima et al. [31, 33] reported is taken into consideration. There is still considerable uncertainty about the stability of the 8-oxodG in urine and all hypotheses should be validated.

Some authors report the stability of the 8-isoprostane in urine as well [35–38], but none of these studies mention its long-term conservation, more than one year, before the analysis. In our study, we showed a slight but significant loss of concentration of this biomarker in urine in the entire sample, as well as in the subsamples stratified by season, warm and cold, when the urine was collected.

The explanation of the decreasing concentrations of the biomarkers stored in urine during long periods can be due to their degradation in it. Further investigations of both the urine biomarkers in an association with environmental, laboratory and human derived predictors, such as the DFC and season, are needed to validate this hypothesis.

The range of two weeks to estimate the reference intervals was chosen based on the medical experience working with RI. Thus, the one-week reference interval might be too brief to track the concentration change of the biomarker, while the one-month reference interval is less sensitive to the variability of the biomarker values.

For the estimation of adjusted reference intervals, the GAMLSS regression analysis proved to be an effective technique providing z-scores (residuals) which can be used to test the adequacy of the model.

## Conclusions

As a main finding of this study, both the OS biomarkers (8-oxodG and 8-isoprostane) should be evaluated in association with the DFC and season when the urine is collected. This is particularly important in large epidemiological studies, when a long-term conservation of urine is required. The (semi)parametric GAMLSS regression analysis is an effective technique that can be used for estimating the adjusted reference intervals of the urinary biomarkers (8-oxodG and 8-isoprostane) in a general adult population.

## Supporting information

S1 Fig The residual distribution of the 8-oxodG in the cold season: The density estimate with rug plot and the quantile-quantile plot for the LOGNO model. (DOCX)

S2 Fig The residual distribution of the 8-oxodG in the warm season: The density estimate with rug plot and the quantile-quantile plot for the LOGNO model. (DOCX)

S3 Fig The residual distribution of the 8-isoprostane in the cold season: The density estimate with rug plot and the quantile-quantile plot for the BCCGo model. (DOCX)

S4 Fig The residual distribution of the 8-isoprostane in the warm season: The density estimate with rug plot and the quantile-quantile plot for the GA model. (DOCX)

S1 Table Comparison between the ‘Standard’, ‘No outlier’s, ‘Cubic splines’ and ‘No outliers + cubic splines’ models for the 8-oxodG and 8-isoprostane during the cold and warm seasons. Standard*—under the standard model here is considered the previously best developed GAMLSS model. df–degrees of freedom for the model fit. BIC–Bayesian Information Criterion.
(DOCX)

S2 Table Predicted reference intervals of the 8-oxodG (ng/mg_creat_) per each 2 weeks (14 days) of the DFC in the cold season. The laboratory processing of the 8-oxodG concentrations in the cold season was started after 36 days of urine storage. DFC—distance from collection (the period from the moment of urine collection and its laboratory processing).
(DOCX)

S3 Table Predicted reference intervals of the 8-oxodG ng/mg_*creat*_ per each 2 weeks (*14 days*) of the DFC in the warm season. The laboratory processing of the 8-oxodG concentrations in the warm season was started after 148 days of urine storage. DFC—distance from collection (the period from the moment of urine collection and its laboratory processing).
(DOCX)

S4 Table Predicted reference intervals of the 8-isoprostane (ng/mg_*creat*_) per each 2 weeks (*14 days*) of the DFC in the cold season. The laboratory processing of the 8-isoprostane concentrations in the cold season was started after 265 days of urine storage. DFC—distance from collection (the period from the moment of urine collection and its laboratory processing).
(DOCX)

S5 Table Predicted reference intervals of the 8-isoprostane (ng/mg_*creat*_) per each 2 weeks (*14 days*) of the DFC in the warm season. The laboratory processing of the 8-isoprostane concentrations in the warm season was started after 230 days of urine storage. DFC—distance from collection (the period from the moment of urine collection and its laboratory processing).
(DOCX)
