# Supplementary figures and images for: Determination of adjusted reference intervals of urinary biomarkers of oxidative stress in healthy adults using GAMLSS models
Source: PLoS One. 2018 Oct 23;13(10):e0206176. doi: 10.1371/journal.pone.0206176 (PMC6198964; doi:10.1371/journal.pone.0206176)

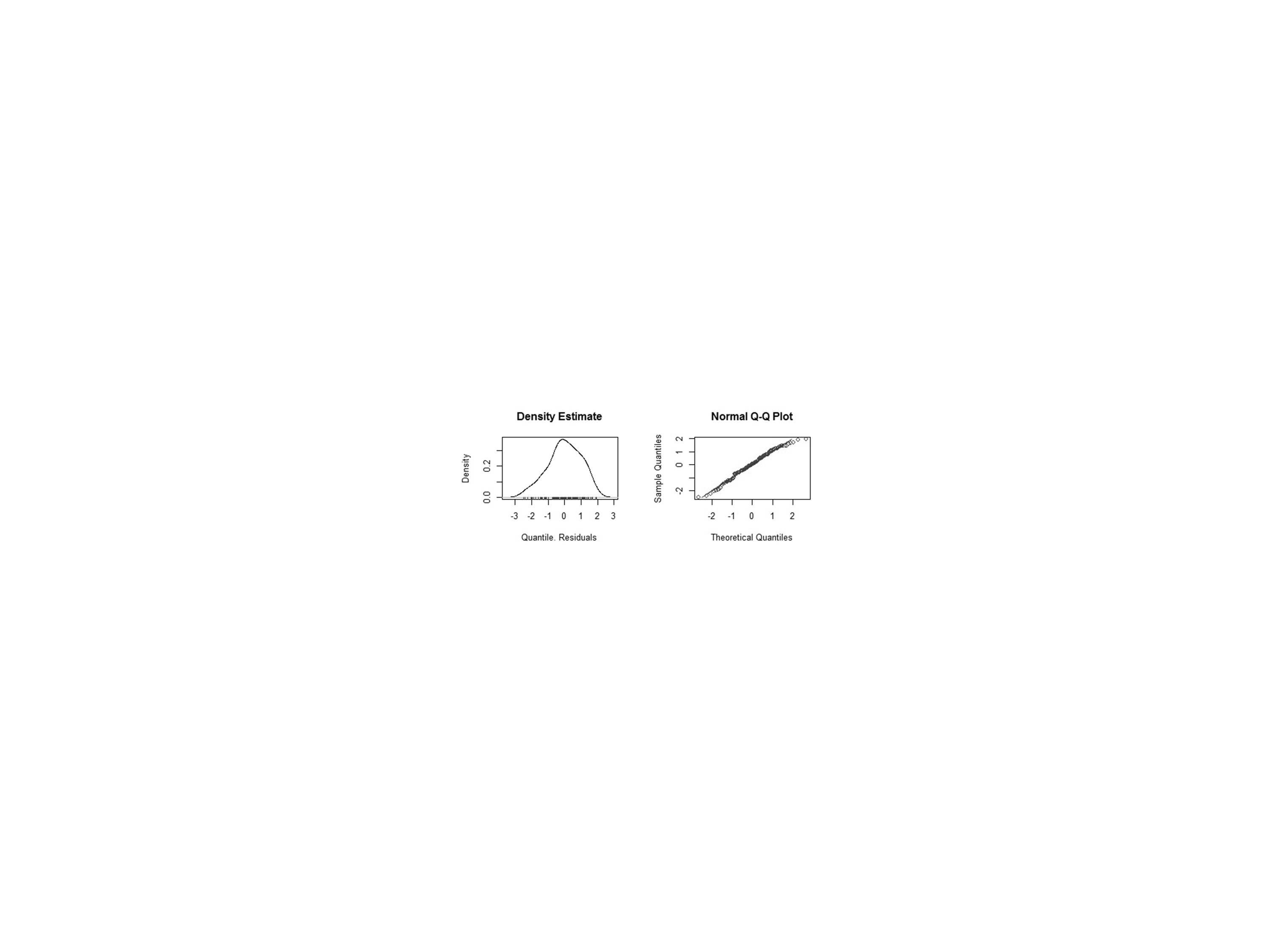


**S1 Fig.**

Supplement: S1 Fig — (DOCX) [file pone.0206176.s001.docx]

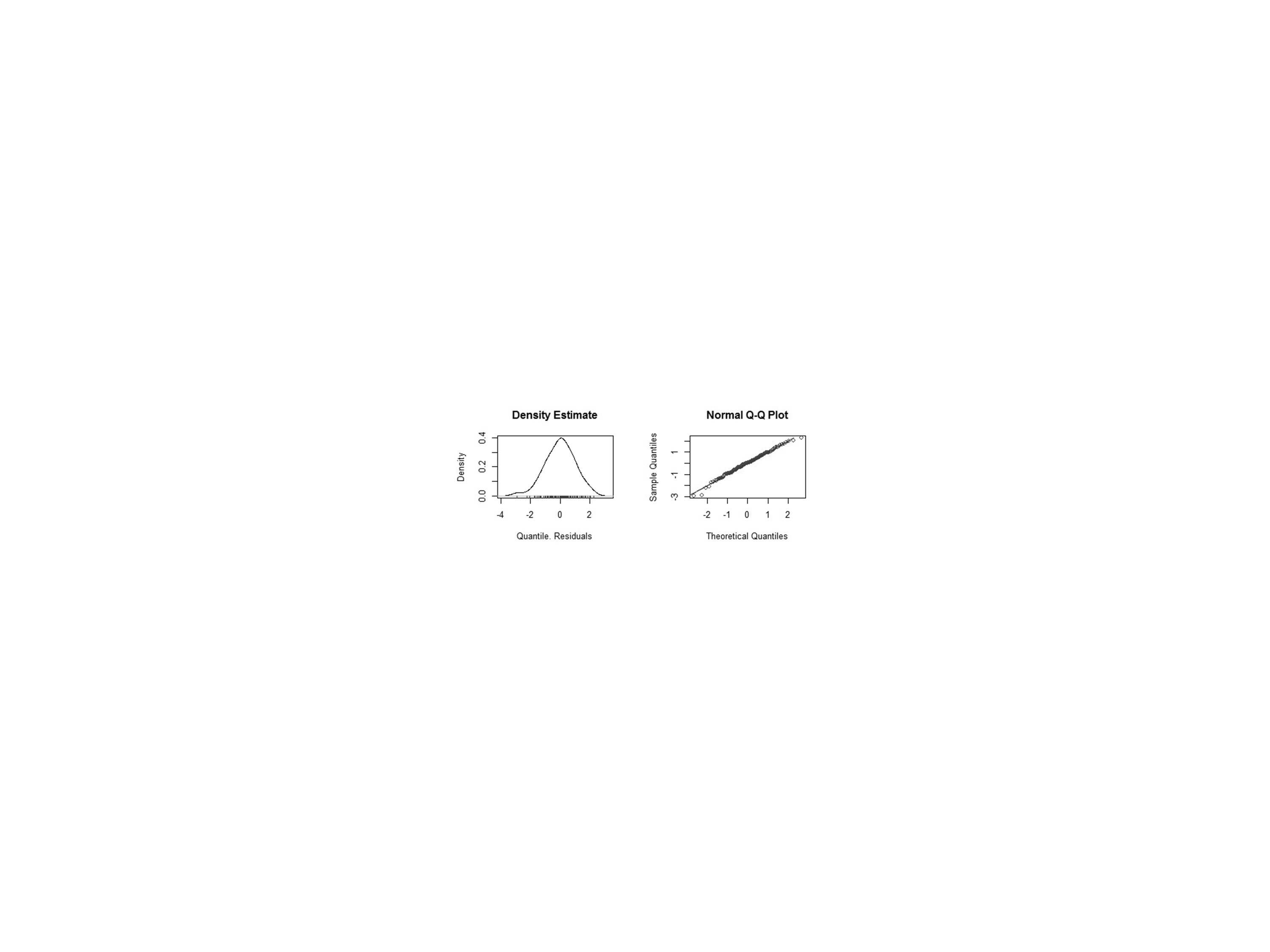
**S2 Fig.**

Supplement: S2 Fig — (DOCX) [file pone.0206176.s002.docx]

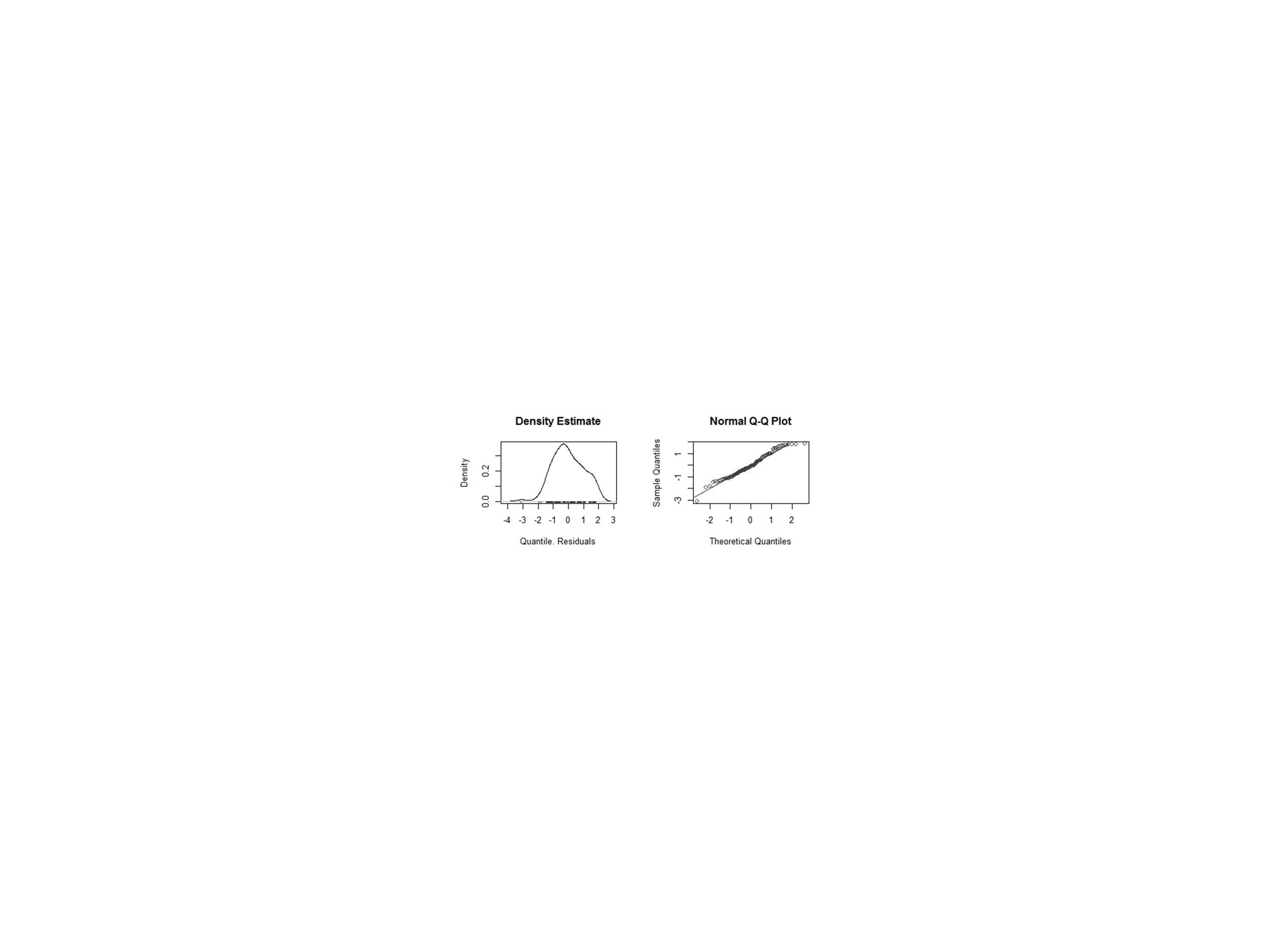


**S3 Fig.**

Supplement: S3 Fig — (DOCX) [file pone.0206176.s003.docx]

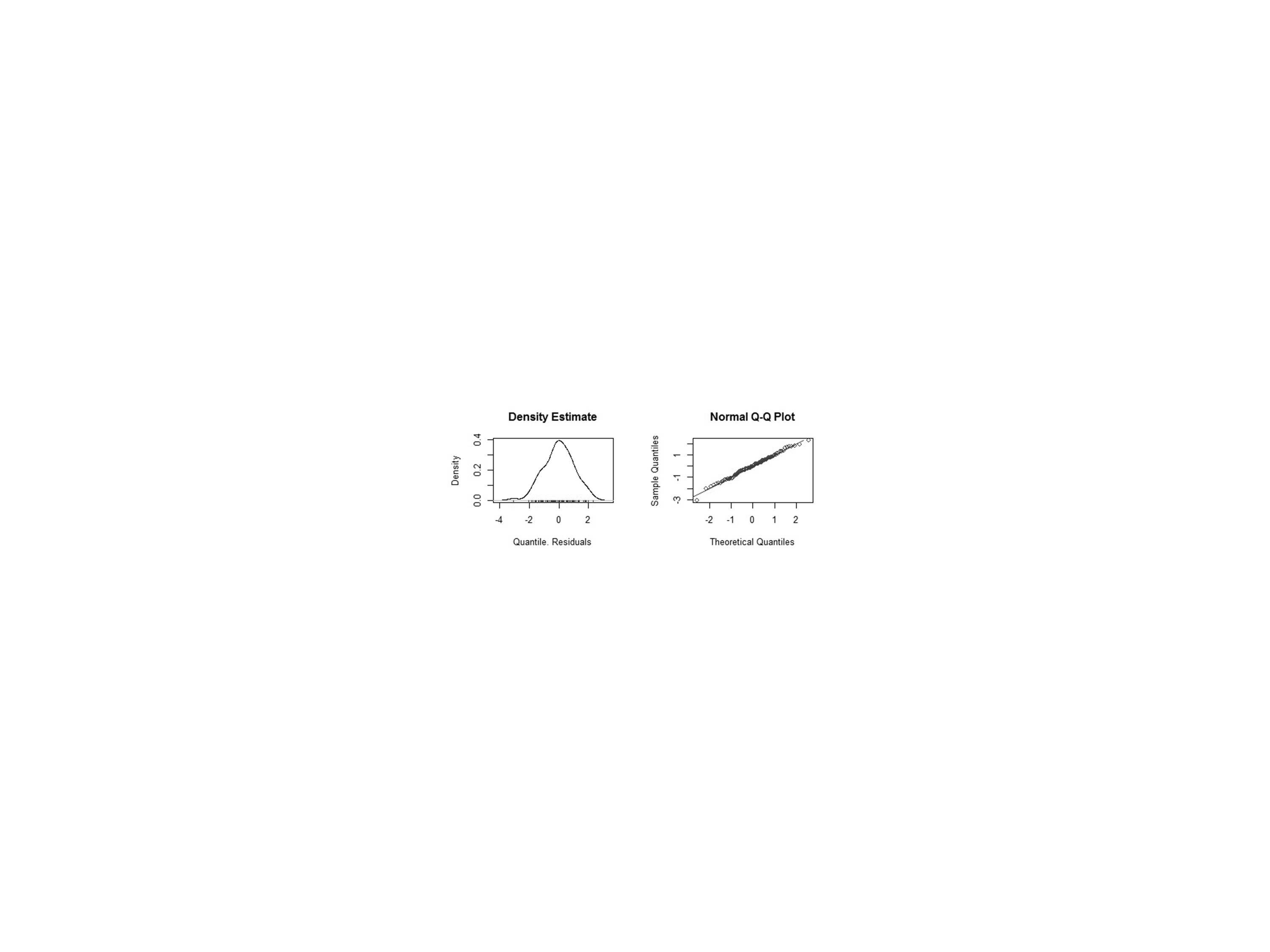
**S4 Fig.**

Supplement: S4 Fig — (DOCX) [file pone.0206176.s004.docx]
