# Supplementary material for: Determination of adjusted reference intervals of urinary biomarkers of oxidative stress in healthy adults using GAMLSS models
Source: PLoS One. 2018 Oct 23;13(10):e0206176. doi: 10.1371/journal.pone.0206176 (PMC6198964; doi:10.1371/journal.pone.0206176)
Supplement: S1 Table — Standard*—under the standard model here is considered the previously best developed GAMLSS model. df–degrees of freedom for the model fit. BIC–Bayesian Information Criterion. (DOCX) [file pone.0206176.s005.docx]

**S1 Table.**

| **Model** | **Distribution** | **df** | **BIC** | **Residuals distribution** | | | |
| --- | --- | --- | --- | --- | --- | --- | --- |
|  |  |  |  | **mean** | **variance** | **coef. of skewness** | **coef. of kurtosis** |
| **8-oxodG. Cold season** | | | | | | | |
| **Standard*** | LOGNO | 5 | 684.90 | 0.0110 | 1.0078 | -0.2199 | 2.6595 |
| **No outliers** | LOGNO | 5 | 631.37 | 0.0045 | 1.0081 | -0.3605 | 2.5457 |
| **Cubic splines** | LOGNO | 6 | 685.20 | 0.0048 | 1.0079 | -0.2701 | 2.5177 |
| **No outliers + Cubic splines** | LOGNO | 6 | 633.41 | 0.0024 | 1.0081 | -0.3892 | 2.4649 |
| **8-oxodG. Warm season** | | | | | | | |
| **Standard*** | LOGNO | 5 | 677.21 | -0.0084 | 1.0083 | -0.0618 | 3.4513 |
| **No outliers** | LOGNO | 5 | 656.51 | -0.0026 | 1.0084 | -0.2983 | 3.1647 |
| **Cubic splines** | LOGNO | 6 | 677.34 | -0.0037 | 1.0083 | -0.0522 | 3.4685 |
| **No outliers + Cubic splines** | LOGNO | 6 | 656.00 | -0.0021 | 1.0084 | -0.2885 | 3.1013 |
| **8-isoprostane. Cold season** | | | | | | | |
| **Standard*** | BCCGo | 6 | 183.17 | -0.0020 | 1.0116 | 0.0093 | 2.2605 |
| **No outliers** | BCCGo | 6 | 169.73 | -0.0024 | 1.0112 | 0.0020 | 2.1098 |
| **Cubic splines** | BCCGo | 7 | 177.59 | -0.0017 | 1.0173 | -0.0074 | 2.2703 |
| **No outliers + Cubic splines** | BCCGo | 7 | 163.77 | -0.0018 | 1.0174 | -0.0168 | 2.1109 |
| **8-isoprostane. Warm season** | | | | | | | |
| **Standard*** | GA | 5 | 235.58 | -0.0002 | 1.0095 | 0.0346 | 2.9073 |
| **No outliers** | GA | 5 | 224.23 | -0.0006 | 1.0081 | -0.0770 | 2.8085 |
| **Cubic splines** | GA | 6 | 238.98 | -0.0003 | 1.0094 | 0.0243 | 2.9271 |
| **No outliers + Cubic splines** | GA | 6 | 227.14 | 0.0018 | 1.0111 | -0.0977 | 2.7759 |
