# Supplementary material for: Determination of adjusted reference intervals of urinary biomarkers of oxidative stress in healthy adults using GAMLSS models
Source: PLoS One. 2018 Oct 23;13(10):e0206176. doi: 10.1371/journal.pone.0206176 (PMC6198964; doi:10.1371/journal.pone.0206176)
Supplement: S2 Table — The laboratory processing of the 8-oxodG concentrations in the cold season was started after 36 days of urine storage. DFC—distance from collection (the period from the moment of urine collection and its laboratory processing). (DOCX) [file pone.0206176.s006.docx]

**S2** **Table.**

|  | **95% Reference Intervals** | |
| --- | --- | --- |
| **DFC, *days*** | **Lower Limit (2.5%)** | **Upper Limit (97.5%)** |
| 36 | 1.68 | 28.85 |
| 50 | 1.59 | 28.28 |
| 64 | 1.51 | 27.73 |
| 78 | 1.43 | 27.20 |
| 92 | 1.36 | 26.69 |
| 106 | 1.29 | 26.19 |
| 120 | 1.22 | 25.70 |
| 134 | 1.15 | 25.23 |
| 148 | 1.09 | 24.77 |
| 162 | 1.04 | 24.33 |
| 176 | 0.98 | 23.89 |
| 190 | 0.93 | 23.47 |
| 204 | 0.88 | 23.07 |
| 218 | 0.83 | 22.67 |
| 232 | 0.79 | 22.29 |
| 246 | 0.74 | 21.92 |
| 260 | 0.70 | 21.56 |
| 274 | 0.66 | 21.21 |
| 288 | 0.63 | 20.87 |
| 302 | 0.59 | 20.54 |
| 316 | 0.56 | 20.22 |
| 330 | 0.53 | 19.91 |
| 344 | 0.50 | 19.61 |
| 358 | 0.47 | 19.32 |
| 372 | 0.45 | 19.04 |
| 386 | 0.42 | 18.77 |
| 400 | 0.40 | 18.50 |
| 414 | 0.37 | 18.25 |
| 428 | 0.35 | 18.00 |
| 442 | 0.33 | 17.76 |
| 456 | 0.31 | 17.53 |
| 470 | 0.29 | 17.30 |
| 484 | 0.28 | 17.09 |
| 498 | 0.26 | 16.88 |
| 512 | 0.25 | 16.68 |
| 526 | 0.23 | 16.48 |
| 540 | 0.22 | 16.29 |
| 554 | 0.20 | 16.11 |
| 568 | 0.19 | 15.94 |
| 582 | 0.18 | 15.77 |
| 596 | 0.17 | 15.61 |
| 610 | 0.16 | 15.45 |
| 624 | 0.15 | 15.30 |
| 638 | 0.14 | 15.16 |
| 652 | 0.13 | 15.03 |
| 666 | 0.12 | 14.89 |
| 680 | 0.12 | 14.77 |
| 694 | 0.11 | 14.65 |
| 708 | 0.10 | 14.54 |
| 722 | 0.10 | 14.43 |
| 736 | 0.09 | 14.33 |
