# Supplementary material for: Determination of adjusted reference intervals of urinary biomarkers of oxidative stress in healthy adults using GAMLSS models
Source: PLoS One. 2018 Oct 23;13(10):e0206176. doi: 10.1371/journal.pone.0206176 (PMC6198964; doi:10.1371/journal.pone.0206176)
Supplement: S3 Table — The laboratory processing of the 8-oxodG concentrations in the warm season was started after 148 days of urine storage. DFC—distance from collection (the period from the moment of urine collection and its laboratory processing). (DOCX) [file pone.0206176.s007.docx]

**S3 Table.**

|  | **95% Reference Intervals** | |
| --- | --- | --- |
| **DFC, *days*** | **Lower Limit (2.5%)** | **Upper Limit (97.5%)** |
| 148 | 0.71 | 21.55 |
| 162 | 0.71 | 21.56 |
| 176 | 0.71 | 21.56 |
| 190 | 0.71 | 21.56 |
| 204 | 0.71 | 21.57 |
| 218 | 0.71 | 21.57 |
| 232 | 0.71 | 21.58 |
| 246 | 0.71 | 21.58 |
| 260 | 0.71 | 21.59 |
| 274 | 0.71 | 21.59 |
| 288 | 0.71 | 21.60 |
| 302 | 0.71 | 21.60 |
| 316 | 0.71 | 21.61 |
| 330 | 0.71 | 21.61 |
| 344 | 0.71 | 21.61 |
| 358 | 0.71 | 21.62 |
| 372 | 0.71 | 21.62 |
| 386 | 0.71 | 21.63 |
| 400 | 0.71 | 21.63 |
| 414 | 0.71 | 21.64 |
| 428 | 0.71 | 21.64 |
| 442 | 0.71 | 21.65 |
| 456 | 0.71 | 21.65 |
| 470 | 0.71 | 21.66 |
| 484 | 0.71 | 21.66 |
| 498 | 0.71 | 21.66 |
| 512 | 0.71 | 21.67 |
| 526 | 0.71 | 21.67 |
| 540 | 0.71 | 21.68 |
| 554 | 0.71 | 21.68 |
| 568 | 0.71 | 21.69 |
| 582 | 0.71 | 21.69 |
| 596 | 0.71 | 21.70 |
| 610 | 0.71 | 21.70 |
| 624 | 0.71 | 21.71 |
| 638 | 0.71 | 21.71 |
| 652 | 0.71 | 21.72 |
| 666 | 0.71 | 21.72 |
| 680 | 0.71 | 21.72 |
| 694 | 0.71 | 21.73 |
| 708 | 0.71 | 21.73 |
| 722 | 0.71 | 21.74 |
| 736 | 0.71 | 21.74 |
| 750 | 0.71 | 21.75 |
| 764 | 0.71 | 21.75 |
