# Supplementary material for: Determination of adjusted reference intervals of urinary biomarkers of oxidative stress in healthy adults using GAMLSS models
Source: PLoS One. 2018 Oct 23;13(10):e0206176. doi: 10.1371/journal.pone.0206176 (PMC6198964; doi:10.1371/journal.pone.0206176)
Supplement: S4 Table — The laboratory processing of the 8-isoprostane concentrations in the cold season was started after 265 days of urine storage. DFC—distance from collection (the period from the moment of urine collection and its laboratory processing). (DOCX) [file pone.0206176.s008.docx]

**S4 Table.**

|  | **95% Reference Intervals** | |
| --- | --- | --- |
| **DFC, *days*** | **Lower Limit (2.5%)** | **Upper Limit (97.5%)** |
| 265 | 0.05 | 4.77 |
| 279 | 0.05 | 4.62 |
| 293 | 0.04 | 4.48 |
| 307 | 0.04 | 4.33 |
| 321 | 0.04 | 4.20 |
| 335 | 0.04 | 4.06 |
| 349 | 0.04 | 3.94 |
| 363 | 0.04 | 3.81 |
| 377 | 0.04 | 3.69 |
| 391 | 0.04 | 3.57 |
| 405 | 0.03 | 3.46 |
| 419 | 0.03 | 3.35 |
| 433 | 0.03 | 3.24 |
| 447 | 0.03 | 3.14 |
| 461 | 0.03 | 3.04 |
| 475 | 0.03 | 2.95 |
| 489 | 0.03 | 2.85 |
| 503 | 0.03 | 2.76 |
| 517 | 0.03 | 2.68 |
| 531 | 0.03 | 2.59 |
| 545 | 0.02 | 2.51 |
| 559 | 0.02 | 2.43 |
| 573 | 0.02 | 2.35 |
| 587 | 0.02 | 2.28 |
| 601 | 0.02 | 2.21 |
| 615 | 0.02 | 2.14 |
| 629 | 0.02 | 2.07 |
| 643 | 0.02 | 2.00 |
| 657 | 0.02 | 1.94 |
| 671 | 0.02 | 1.88 |
| 685 | 0.02 | 1.82 |
| 699 | 0.02 | 1.76 |
| 713 | 0.02 | 1.71 |
| 727 | 0.02 | 1.65 |
| 741 | 0.02 | 1.60 |
