# Supplementary material for: Determination of adjusted reference intervals of urinary biomarkers of oxidative stress in healthy adults using GAMLSS models
Source: PLoS One. 2018 Oct 23;13(10):e0206176. doi: 10.1371/journal.pone.0206176 (PMC6198964; doi:10.1371/journal.pone.0206176)
Supplement: S5 Table — The laboratory processing of the 8-isoprostane concentrations in the warm season was started after 230 days of urine storage. DFC—distance from collection (the period from the moment of urine collection and its laboratory processing). (DOCX) [file pone.0206176.s009.docx]

**S5 Table.**

|  | **95% Reference Intervals** | |
| --- | --- | --- |
| **DFC, *days*** | **Lower Limit (2.5%)** | **Upper Limit (97.5%)** |
| 230 | 0.06 | 6.54 |
| 244 | 0.06 | 6.40 |
| 258 | 0.06 | 6.27 |
| 272 | 0.06 | 6.14 |
| 286 | 0.06 | 6.01 |
| 300 | 0.06 | 5.88 |
| 314 | 0.06 | 5.76 |
| 328 | 0.06 | 5.64 |
| 342 | 0.05 | 5.52 |
| 356 | 0.05 | 5.41 |
| 370 | 0.05 | 5.29 |
| 384 | 0.05 | 5.18 |
| 398 | 0.05 | 5.07 |
| 412 | 0.05 | 4.97 |
| 426 | 0.05 | 4.86 |
| 440 | 0.05 | 4.76 |
| 454 | 0.05 | 4.66 |
| 468 | 0.05 | 4.56 |
| 482 | 0.04 | 4.47 |
| 496 | 0.04 | 4.38 |
| 510 | 0.04 | 4.28 |
| 524 | 0.04 | 4.20 |
| 538 | 0.04 | 4.11 |
| 552 | 0.04 | 4.02 |
| 566 | 0.04 | 3.94 |
| 580 | 0.04 | 3.86 |
| 594 | 0.04 | 3.77 |
| 608 | 0.04 | 3.70 |
| 622 | 0.04 | 3.62 |
| 636 | 0.04 | 3.54 |
| 650 | 0.03 | 3.47 |
| 664 | 0.03 | 3.40 |
| 678 | 0.03 | 3.33 |
| 692 | 0.03 | 3.26 |
| 706 | 0.03 | 3.19 |
| 720 | 0.03 | 3.12 |
| 734 | 0.03 | 3.06 |
| 748 | 0.03 | 2.99 |
| 762 | 0.03 | 2.93 |
